# University of Louisville

**Published:** 1853-03

**Authors:** 


### THE WESTERN JOURNAL

THIRD SERIES.—VOL. XI.—NO. III.

LOUISVILLE, MARCH 1, 1853.

### UNIVERSITY OF LOUISVILLE.

The Annual Commencement in the Medical Department of the University of Louisville took place on Thursday evening, the 3d of March, on which occasion eighty-seven gentlemen received the degree of M. D., in ordinary. The *ad-Eundem* degree was conferred upon one, and two received the honorary degree. A catalogue of the graduates will be appended to this number of the Journal. The annual number of the graduates in the University has varied but little for several years past. Once it rose above a hundred, but the number has generally been about ninety. The customary valedictory address was delivered by Prof. Rogers, and was prepared with unusual care. Its topics were not those which are generally chosen on such occasions, but it treated of the Science of Medicine as it is, somewhat in the style in which Dr. Forbes exhibited it in his far-famed essays. A critic might have detected in it some points for animadversion, but we take more pleasure in commending it for its learning, its frank spirit, and its philosophical tone, all of which it displayed in an uncommon degree.

